# Implementing HPV testing in 9 Latin American countries: The laboratory perspective as observed in the ESTAMPA study

**DOI:** 10.3389/fmed.2022.1006038

**Published:** 2022-11-17

**Authors:** Mary Luz Rol, María Alejandra Picconi, Annabelle Ferrera, Gloria Inés Sánchez, María de la Luz Hernández, Joana Lineros, Ana Peraza, Marisol Brizuela, Laura Mendoza, Pamela Mongelós, Yessy Cabrera, Margarita Rodríguez de la Peña, Rita Mariel Correa, Carolina Terán, Deisy Colque Reynaga, Laura García, Arianis Tatiana Ramírez, Pilar Hernández-Nevarez, Franco Doimi, María Ramón, Javier Arias-Stella, Michael Zúñiga, Verónica Villagra, María Liz Bobadilla, Lucía Cardinal, Joan Valls, Eric Lucas, Armando Baena, Laura Fleider, Gino Venegas, Aurelio Cruz-Valdez, Guillermo Rodríguez, Alejandro Calderón, Carolina Wiesner, Silvana Luciani, Nathalie Broutet, Rolando Herrero, Maribel Almonte

**Affiliations:** ^1^Early Detection, Prevention and Infections Branch, International Agency for Research on Cancer, Lyon, France; ^2^Instituto de Enfermedades Infecciosas ANLIS-Malbrán, Buenos Aires, Argentina; ^3^Instituto de Investigaciones en Microbiología, Universidad Nacional Autónoma de Honduras, Tegucigalpa, Honduras; ^4^Grupo de Infección y Cáncer, Universidad de Antioquia, Medellín, Colombia; ^5^SMS-Oncology, Amsterdam, Netherlands; ^6^Instituto Nacional de Cancerología, Bogotá, Colombia; ^7^Caja Costarricense de Seguro Social (CCSS), Región Pacífico Central, San José, Costa Rica; ^8^Instituto de Investigaciones en Ciencias de la Salud, Universidad Nacional de Asunción, San Lorenzo, Paraguay; ^9^Hospital Nacional Profesor Alejandro Posadas, El Palomar, Argentina; ^10^Facultad de Medicina, Universidad Mayor, Real y Pontificia de San Francisco Xavier de Chuquisaca, Sucre, Bolivia; ^11^Laboratorio de Biología Molecular, Departamento de Patología Clínica, Centro Hospitalario Pereira Rossell, Montevideo, Uruguay; ^12^Instituto de Salud Pública de México, Morelos, Mexico; ^13^Laboratorio de Patología Oncológica SAC, Lima, Peru; ^14^Instituto de Patología y Biología Molecular, Lima, Peru; ^15^Agencia Costarricense de Investigaciones Biomédicas (ACIB), Fundación Inciensa, San José, Guanacaste, Costa Rica; ^16^Laboratorio Central de Salud Pública, Asunción, Paraguay; ^17^Hospital de Clínicas José de San Martín, Buenos Aires, Argentina; ^18^Clínica Angloamericana, Lima, Peru; ^19^Liga contra el Cáncer, Lima, Peru; ^20^Comisión Honoraria de Lucha Contra el Cáncer, Montevideo, Uruguay; ^21^Pan American Health Organization (PAHO), Washington, DC, United States; ^22^Department of Sexual and Reproductive Health and Research, World Health Organization, Geneva, Switzerland

**Keywords:** HPV testing, HPV testing implementation, readiness and continuity capacity, ESTAMPA study, cervical cancer screening, Latin America

## Abstract

**Background:**

Replacement of cytology screening with HPV testing is recommended and essential for cervical cancer elimination. HPV testing for primary screening was implemented in 12 laboratories within 9 Latin American countries, as part of the ESTAMPA cervical cancer screening study. Our observations provide information on critical operational aspects for HPV testing implementation in diverse resource settings.

**Methods:**

We describe the implementation process of HPV testing in ESTAMPA, focusing on laboratory aspects. We assess the *readiness* of 12 laboratories to start HPV testing and their *continuity capacity* to maintain good quality HPV testing until end of recruitment or up to December 2021. *Readiness* was based on a checklist. Information from the study database; regular meetings and monitoring visits; and a questionnaire on laboratory operational aspects sent in May 2020 were used to assess *continuity capacity*. Compliance with seven basic requirements (readiness) and eight continuity requirements (continuity capacity) was scored (1 = compliant, 0 = not compliant) and totaled to classify *readiness* and *continuity capacity* as very limited, limited, moderate or high. Experiences, challenges, and enablers of the implementation process are also described.

**Results:**

Seven of 12 laboratories had *high readiness*, three *moderate readiness*, and of two laboratories new to HPV testing, one had *limited readiness* and the other *very limited readiness*. Two of seven laboratories with *high readiness* also showed *high continuity capacity*, one *moderate continuity capacity*, and the other four showed *limited continuity capacity* since they could not maintain good quality HPV testing over time. Among three laboratories with *moderate readiness*, one kept *moderate continuity capacity* and two reached *high continuity capacity.* The two laboratories new to HPV testing achieved *high continuity capacity*. Based on gained expertise, five laboratories have become part of national screening programs.

**Conclusion:**

*High readiness* of laboratories is an essential part of effective implementation of HPV testing. However, *high readiness* is insufficient to guarantee HPV testing *high continuity capacity*, for which a “culture of quality” should be established with regular training, robust monitoring and quality assurance systems tailored to local context. All efforts to strengthen HPV laboratories are valuable and crucial to guarantee effective implementation of HPV-based cervical screening.

## Introduction

More than 600,000 new cases and 300,000 cervical cancer deaths occur every year; over 90% of these are in low-income and middle-income countries (LMIC) (1).

Cytology-based screening has successfully reduced cervical cancer rates in places where it has been systematically implemented (2). However, cytology has limited and variable sensitivity, requiring frequent repetition to reach an acceptable level of precancerous lesions detection (3, 4). Frequent cytology screening has not been feasible in most LMIC, where coverage is generally low, and follow-up of women with abnormal cytology and treatment of detected lesions is very limited (3, 5).

Persistent infection with high-risk HPV is the leading cause of cervical cancer (6). Several molecular techniques are available to detect HPV DNA and can be used in primary screening (7, 8). There is overwhelming worldwide evidence that HPV testing is more effective than cytology in identifying women at greater risk of precancerous cervical lesions (9–13). HPV testing is objective, can be automated, and can be done using self-collected samples with potential to increase screening coverage (14–17). In addition, the high negative predictive value of HPV testing allows extension of the screening interval (in comparison to the 3 years interval when using cytology) (18, 19), facilitating screening and treatment coverage in limited-resource settings.

In the World Health Assembly (20) adopted a global strategy for eliminating cervical cancer as a public health problem. In order to reach elimination by the end of the century, countries should achieve full vaccination of 90% of girls by age 15, screening of 70% of women twice by age 35 and 45 with a high-performance test, and treatment of 90% of women with cervical disease (precancer or invasive cervical cancer) (21). In 2021, WHO published cervical cancer screening and treatment guidelines recommending the use of HPV DNA detection in a screen and treat approach or a screen, triage and treat approach starting at age 30 every 5 to 10 years for the general population, and using HPV DNA detection in a screening, triage and treat approach starting at age 25 every 3 to 5 years for women living with HIV (22). As evidence-based cervical cancer screening and treatment interventions are available, it is time for country-driven implementation research to understand how to implement and scale-up HPV-based cervical screening (23, 24). In fact, reports from countries in Latin America that are replacing cytology with HPV testing at the national level or in pilots suggest that HPV-based cervical screening implementation is very challenging, and demands extensive planning of activities across the screening care continuum including preparation of laboratory facilities and training of personnel for HPV testing before scaling-up (25–29).

Several tools are available to support countries with the implementation of HPV testing within cervical cancer screening (30–33). In particular, the step-by-step guide on introducing and scaling up HPV within a comprehensive program of prevention and control of cervical cancer (33), intends to offer practical guidance to program managers once the decision to introduce HPV testing in their national cervical cancer prevention program has been made. Guidance covers three main domains: planning, implementation, and monitoring/scaling up of HPV testing in primary screening. Once planning is completed, the preparation of an implementation roadmap is recommended, to establish or strengthen quality management systems in laboratories, to define the procurement process and to use indicators to monitor the progress of the implementation. This is key to consolidate a screening platform for scaling up and final adoption of the screening strategy at national level. Using several features of this step-by-step guide, here we describe the HPV testing implementation process, including monitoring and evaluation of HPV testing performance over time of 12 laboratories in Latin America participating in the ESTAMPA study (NCT01881659) (34).

## Materials and methods

ESTAMPA is a multicentric study of cervical cancer screening with HPV testing conducted in 12 study centers’ laboratories (SC1-12) in nine countries in Latin America. The study aims to evaluate the performance of different techniques and approaches to triage HPV positive women and to inform on how best to implement affordable and sustainable HPV-based screening programs in LMIC.

The study protocol has been previously published (34). Briefly, women aged 30 to 64 years old were invited for cervical cancer screening with HPV testing and, following country guidelines, also cytology. Women who consented to participate underwent pelvic examination, and samples were collected using Cervex brushes (Papette, Wallach, USA) that were washed in PreservCyt medium (PC) (Hologic, USA) for HPV testing and cytology.

Samples were tested for HPV DNA detection using either *Digene* HC2 HPV DNA test (QIAGEN, USA) or COBAS HPV test (Roche, Switzerland). After HPV testing, aliquots of PC were prepared for future molecular triage. *Digene* HC2 sample conversion kit was used for conversion of samples collected on PC before HC2 HPV testing. HPV (HC2 or COBAS) was done following manufacturer instructions and ESTAMPA standard operating procedures (SOPs). For quality control (QC) of HPV testing, in some laboratories, about 10% of samples tested with HC2 or COBAS were retested either at the same laboratory using a different HPV technique or at and international hub with COBAS. In one center, samples from the first 900 recruited women were stored in the Digene Standard Transport Medium (STM) and subsequently tested with HC2. In this center, a sub-study evaluating the impact of operational factors on HPV positivity of HPV assays including HC2, COBAS and APTIMA (Hologic, USA) was conducted and COBAS results were assumed as QC of HC2 and APTIMA (35).

Women with abnormal cytology or positive HPV results (including QC HPV test) were referred to colposcopy, 2-3 biopsies were collected from any observed lesions, and women diagnosed with cervical intraepithelial neoplasia (CIN) grade 2 or worse (CIN2 +) were treated with LLETZ. At this colposcopy visit, a cervical sample was collected for HPV testing and other molecular triage tests. Women with negative colposcopy or histology showing lesions less severe than CIN2 (< CIN2) were invited for a follow-up visit at 18 months for a second HPV screen to complete ascertainment of disease. Women positive for HPV at this visit were referred to colposcopy and clinically managed as needed.

Overall, women could have one, two, three or four HPV tests during the study, depending on their screening results and associated study visits (initial screening, colposcopy and 18months follow-up) and on the sample being selected for QC.

### Steps to implement HPV testing in 12 ESTAMPA laboratories

**FIGURE 1:**
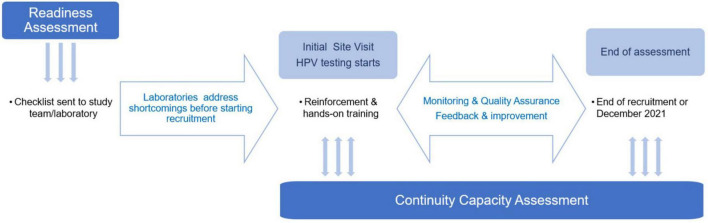
Steps followed in ESTAMPA for HPV testing implementation.

The main steps carried out to implement HPV testing in ESTAMPA laboratories (Figure 1) are described as follows.

(1) Assessment of *readiness* for HPV testing implementation in the laboratory. Before launching the study, local principal investigators willing to take part, were asked to identify a laboratory with adequate facilities as to carry out HPV testing for primary screening. Laboratory-based investigators could propose their own facilities or others thought more suitable for future participation in national HPV-based cervical screening program.

Next, a checklist (Supplementary Material 1) was used to assess the existing installed capacity of each laboratory. As the study was launched early on in the introduction of HPV testing in primary screening (December 2012), laboratories were asked about the availability of HPV equipment in the laboratory; whether collection medium and HPV reagents were registered in the country and if not, whether investigators had experience with importing and customs; and whether additional HPV testing consumables specifically selected for the study were available in the laboratory or the country.

Laboratories were also asked whether they had personnel to perform the test; and whether they had established logistics for transport and storage of samples, management of reagents validity and delivery of results. We did not request specific arrangements for external quality assurance (EQA) in this checklist, but we proposed different QA measures as the study progressed.

Based on this initial assessment, each study center developed a road map to address limitations and joined the project when ready to launch recruitment.

(2) Initial site visit to start recruitment and HPV testing. The study covered the cost of HPV reagents and additional consumables where needed. Procurement was done internationally or in-country, depending on availability of local distributors and registration of products. Once consumables were in place (usually for the first 500-1,000 tests) and laboratories were ready, a site visit by IARC researchers to launch the ESTAMPA study was organized.

During this initial visit, training on the study protocol, study’s SOPs and good clinical practices was given to all study personnel over 1-2 days, followed by real-life recruitment. In parallel, the selected HPV testing laboratory was visited to verify that conditions were ready to start testing. When enough samples for a full- or a half-testing batch (3-7 days depending on local arrangements) had been collected, additional training on the study protocol, laboratory SOPs and overall sample handling from reception at the laboratory to delivery of results, was provided to all laboratory personnel.

Next, using recently collected samples, hands-on training was given to 1-2 technicians on: (i) Running HPV testing if no previous experience, (ii) Performing conversion of samples collected in PC if HC2 testing was used, (iii) Preparing aliquots from PC vials, and (iv) Recording results on the study database. Lead investigators at each center made arrangements to repeat this training as needed when new laboratory personnel joined the study.

As the study progressed initial and monitoring site visits were done by a team mostly composed by local researchers selected from study centers already recruiting with support from ESTAMPA researchers.

(3) Assessment of *continuity capacity* of laboratories to perform good quality HPV testing. Laboratories’ continuity capacity was assessed throughout the study. Sources of information used for the assessment included: minutes from meetings with local investigators and laboratory managers, reports from monitoring visits, data provided by the study centralized web-system, and from a questionnaire on laboratory operational aspects (Supplementary Material 2) sent to laboratory managers in May 2020.

As laboratories joined the study, implementation of quality assurance processes was recommended, such as providing regular training to technicians on HPV testing and study’s SOPs, retesting of a subset of samples and annual subscription to EQA. The study covered the first year EQA fees for new laboratories which were encouraged to ensure funding for next subscriptions.

The centralized web-system specifically developed for the study allowed regular checking on the progress of recruitment, the number of HPV tests carried out (at enrolment, colposcopy, and 18 months), the speed of uploading results, and statistics on clinical activities. Thus, the web-system provided effective indicators of the HPV testing capacity of laboratories, such as the volume of HPV testing and the HPV positivity over time. As over 75% of samples tested in each laboratory were collected at the initial screening visit, the number of recruited women per study center was used as a proxy of HPV testing volume, and the positivity of HPV testing at the time of enrolment was used as a proxy of overall HPV positivity, independently of the HPV detection technique used (HC2, COBAS). Based on previously reported HPV prevalence among Latin American women (36–38), we assumed that HPV positivity values reported by laboratories should be between 12.5 and 15%. HPV positivity was regularly tabulated by center and was graphically assessed by plotting the HPV positivity with 95% confidence intervals (using exact binominal distribution and the R statistical package for computations). If any laboratory showed HPV positivity below 10% or above 16% in two consecutive runs, on any assessment, a full data inspection across the screening process (i.e., sample collection, HPV testing and laboratory activities, clinical management, data management) was done and whenever necessary was followed by a site visit to offer refresher training or suggest corrective measures.

The questionnaire sent to laboratory managers requested current details on HPV equipment status, annual subscriptions to EQA, training on HPV testing for current and new technicians, preparedness for procurement process of HPV and related laboratory supplies, availability of tracking systems for HPV reagents and laboratory supplies and for samples transport and storage and turnaround time of HPV results.

As recruitment progressed, many logistic activities were smoothly handled over to local investigators, including procurement of reagents and consumables if they became available in the country, and responsibility for maintaining adequate stocks. This transition facilitated implementation or expansion of HPV-based cervical screening programs.

All collected data were used to assess whether laboratories were able to continue performing good quality HPV testing over time, from the first batch of testing up until end of recruitment or December 2021.

### Evaluation of laboratories’ readiness and continuity capacity for HPV testing implementation

Collected data from the readiness and continuity capacity assessments were summarized in six main domains: characteristics of the laboratory facility, personnel, procurement of reagents and consumables, tracking systems for reagents expiry and supplies, logistics for transport and storage and delivery of testing results. Responses to the initial checklist were classified into seven basic requirements (within the above six domains) and were used to assess the laboratory *readiness* for HPV implementation. Monitoring, and operational questionnaires classifying eight continuity requirements (within the six domains), were used to assess the *continuity capacity* of laboratories to perform good quality HPV testing over time.

Each requirement was scored as compliant (value = 1) or non-compliant (value = 0), and values were summed up to generate a total score (range = 0-7 for readiness and 0-8 for continuity capacity). Using the scores, the readiness of laboratories to start HPV testing at the time of initial assessment was classified as *very limited* (score ≤ 3), *limited* (score = 4-5), *moderate* (score = 6) and *high* (score = 7). Similarly, the continuity capacity of laboratories to perform HPV testing over time was classified as *very limited* (score ≤ 3), *limited* (score = 4-5), *moderate* (score = 6-7) and *high* (score = 8) (Tables 1, 2).

**TABLE 1:** *Readiness* of laboratories based on compliance (1 = complied, 0 = not complied) to 7 essential requirements.

Study center/Laboratory (SC)	Laboratory facility	Personnel	Procurement		Tracking systems	Transport and storage	Delivery of results	Classification	
							
	HPV testing equipment available in the laboratory (HC2 or COBAS)	Personnel designated to perform HPV testing	Collection medium, HPV reagents registration in the country. If not, experience with importing/customs	Additional HPV testing consumables available in the laboratory or in the country*	Laboratory management of reagents validity logistics	Logistics for transport and storage of samples	Logistics for delivery of results (standard report template, time and route to reach women)	Total score of HPV testing requirements implemented	Readiness level
SC1	1	1	1	1	1	1	1	**7**	High
SC2	1	1	1	1	1	1	0	**6**	Moderate
SC3	1	1	1	1	1	1	0	**6**	Moderate
SC4	0	1	1	1	1	1	1	**6**	Moderate
SC5	1	1	1	1	1	1	1	**7**	High
SC6	1	1	1	1	1	1	1	**7**	High
SC7	0	0	1	1	1	1	1	**5**	Limited
SC8	1	1	1	1	1	1	1	**7**	High
SC9	1	1	1	1	1	1	1	**7**	High
SC10	1	1	1	1	1	1	1	**7**	High
SC11	1	1	1	1	1	1	1	**7**	High
SC12	0	0	1	1	0	1	0	**3**	Very limited

*Other laboratory consumables specific and standard for the study. Readiness classification according to scores: 7 = high readiness, 6 = moderate readiness, 4-5 = limited readiness, ≤ 3 = very limited.

**TABLE 2:** *Continuity capacity* of laboratories based on compliance (1 = complied, 0 = not complied) to 8 continuity requirements.

Study center/Laboratory (SC)	Laboratory facility		Personnel	Procurement		Tracking systems	Transport and storage	Delivery of results	Classification	
							
	Maintenance of HPV equipment (regular inspection, calibration)	Regular subscription to external quality assurance for HPV testing	Regular training on HPV testing & laboratory SOPs	Procurement ability for timely purchase of consumables	Appropriate coordination for importation of supplies	Tracking system for HPV reagents and other consumables	Tracking system for transport and storage of samples and aliquots	Turnaround of HPV testing results within one month	Total score of HPV testing requirements sustained over time	Continuity capacity level
SC1	1	1	1	1	1	1	1	1	**8**	High
SC2	1	1	1	1	1	1	1	0	**7**	Moderate
SC3	1	1	1	1	1	1	1	1	**8**	High
SC4	1	1	1	1	1	1	1	1	**8**	High
SC5	1	1	1	1	1	1	1	1	**8**	High
SC6	1	1	1	1	1	1	1	0	**7**	Moderate
SC7	1	1	1	1	1	1	1	1	**8**	High
SC8	1	0	1	1	0	1	1	0	**5**	Limited
SC9	1	0	1	1	1	0	1	0	**5**	Limited
SC10	1	1	1	1	0	0	1	0	**5**	Limited
SC11	0	1	1	1	1	0	1	0	**5**	Limited
SC12	1	1	1	1	1	1	1	1	**8**	High

Continuity capacity classification according to scores: 8 = high, 6-7 = moderate, 4-5 = limited and ≤ 3 = very limited.

## Results

**FIGURE 2:**
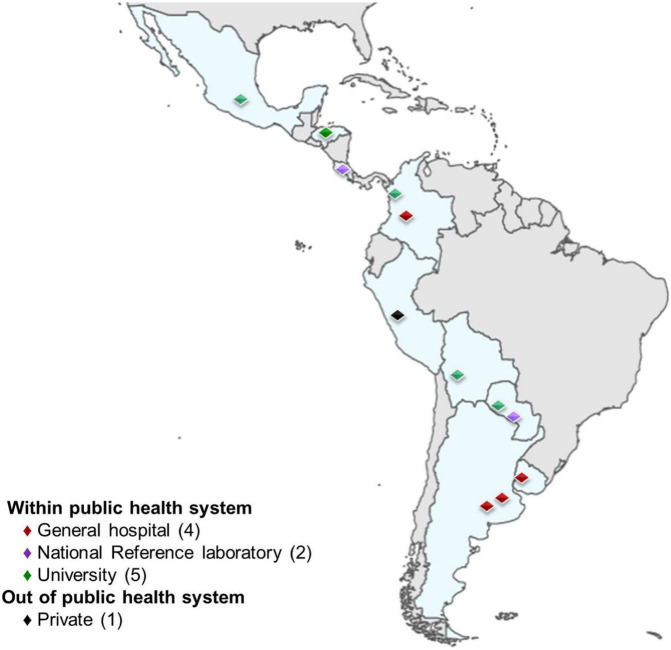
Laboratory location and health system affiliation.

Twelve study centers with corresponding HPV laboratories, located in nine Latin American countries (Argentina, Bolivia, Colombia, Costa Rica, Honduras, Mexico, Paraguay, Peru and Uruguay) participated in the ESTAMPA study. Eleven laboratories were part of public systems (4 were based in general hospitals, 2 were national-referral laboratories, 5 were university-based), and one offered service under a private system. Six laboratories had staff with experience in detecting HPV for clinical diagnosis and research at the initial assessment. Two of the remaining laboratories had experience in detecting HPV for research only, and two had experience in HPV detection for diagnosis but not for research. One laboratory was set up in a regional hospital, but the study team was based on a high-level expertise (diagnostics and research) HPV reference laboratory. Finally, one laboratory did not have previous experience in HPV detection or any other molecular diagnostics (Figure 2).

Despite differences in recruitment targets (ranging from 500 to 10,000) and year when recruitment and HPV testing started (2012 to 2017), among 42,502 women with valid HPV results recruited until December 2021, the overall yearly HPV positivity consistently ranged between 12 and 16% over time (mean HPV positivity: 14.1%, 95CI: 13.8-14.4) (Supplementary Figure 1).

### Compliance with readiness requirements

Table 1 summarizes the compliance with the seven readiness requirements per laboratory. Nine of the twelve laboratories had equipment for HPV detection (8 used HC2, 1 used Cobas). Procurement of equipment for the remaining three laboratories was dependent on when the local team agreed to participated in ESTAMPA and the availability of local funding. In study center 7 (SC7), the principal investigator who led an international reference HPV laboratory, obtained a Cobas machine under a loan for study purposes, which was installed in a regional hospital, as the national cervical screening program had plans to implement HPV testing there. The SC7 team trained, supervised, and supported the newly laboratory throughout the study. HC2 equipment was procured by the central team in 2014 for the laboratory working with study center 4 (SC4), while study center 12 (SC12) secured local funding for HC2 equipment, although political and administrative complexities meant final procurement did not happen until 2018. Regardless of previous experience on HPV detection techniques, all laboratories except the new ones, SC7 and SC12, had personnel allocated to perform HPV testing at the time of initial assessment.

As the study provided all relevant consumables at the start, centers did not need to engage in direct procurement activities. However, all teams and particularly laboratory leads demonstrated awareness of current local regulations for purchasing in-country, and the ability to deal with importation of non-registered in-country consumables or to negotiate prices of testing kits and machine loaning as SC7.

All laboratories had established logistics for transporting and storage samples (as per protocol), only (SC12) did not have a system to control the validity of laboratory reagents, and in addition to SC2 and SC3 did not have a system in place to return results to women, did not have a standard report template nor instructions on how to share results with participants.

In summary, seven laboratories (SC1, SC5, SC6, SC8, SC9, SC10, and SC11) had *high readiness* at initial assessment and three (SC2, SC3 and SC4) had *moderate readiness* with only one essential requirement not fulfilled. Of two laboratories totally new to HPV testing, the one (SC7) supported by a high-level laboratory had *limited readiness* and the other one (SC12) *very limited readiness*.

### Compliance with continuity capacity requirements

Table 2 summarizes the compliance with the eight continuity capacity requirements per laboratory. All laboratories had regular maintenance including cleaning and calibration of their HPV testing equipment except for SC11 which was not serviced on time and testing had to stop, leading to severe delays in processing samples. Six laboratories (SC1, SC2, SC5, SC6, SC7, and SC10) that were already performing HPV testing (mainly for research) at initial assessment, had established EQA systems and maintained regular participation in either the College of Pathologists (CAP) scheme or the Quality Control for Molecular Diagnostics (QCMD). The study covered the first subscription payment for other four laboratories (SC3, SC4, SC11, and SC12), but only one (SC11) maintained the subscription using their own funds afterward, and two laboratories (SC8 and SC9) never subscribed to EQA during the assessment period. In parallel to these schemes, all laboratories were encouraged to repeat testing of 10% of samples, preferably with a different HPV detection platform or in a different laboratory.

All laboratories offered appropriate training in HPV testing and laboratory study SOPs to new laboratory personnel, even if they were not assigned to HPV testing and gained or improved the ability to ensure timely procurement of HPV reagents and other consumables.

Laboratories developed procurement plans, independently of whether the planning only involved preparing paperwork for customs clearance in advance or dealing with local distributors or purchasing the consumables (with study’s funds, health authorities funds or research granted competitive funds). However, two laboratories (SC8, SC10) had difficulties coordinating supplies’ importation all over recruitment.

Laboratory management systems (manual or digital) for tracking of reagents expiry and handling of samples (transport and storage) were incorporated or improved by all laboratories. However, three laboratories (SC9, SC10, and SC11) needed the central team to send regular reminders to verify expiration dates throughout the study.

Six laboratories struggled to consistently deliver results within one month. Four of them (SC8, SC9, SC10, and SC11) had additional challenges linked to procurement of consumables, while the delays for the other two laboratories (SC2 and SC6) were largely related to the study setting. SC2 recruited in a remote location, and samples were shipped by air only 1-2 times per month. SC6 recruited in several centers concurrently in order to reach its recruitment target of 10,000 women. This meant that samples arrived at both laboratories in large batches and accumulated, causing delays with processing and turnaround of HPV results. In such cases, different measures were applied when to guarantee adequate follow-up of screen-positives (e.g., testing in another laboratories, using cytology results for referral to or offering colposcopy to those with delayed results).

Overall, six laboratories (SC1, SC3, SC4, SC5, SC7, and SC12) showed *high continuity capacity* as they fulfilled all criteria; two (SC2 and SC6) had *moderate continuity capacity* having only not fulfilled one requirement and four (SC8, SC9, SC10 and SC11) showed *limited continuity capacity*.

### Relation between readiness and continuity capacity

**FIGURE 3:**
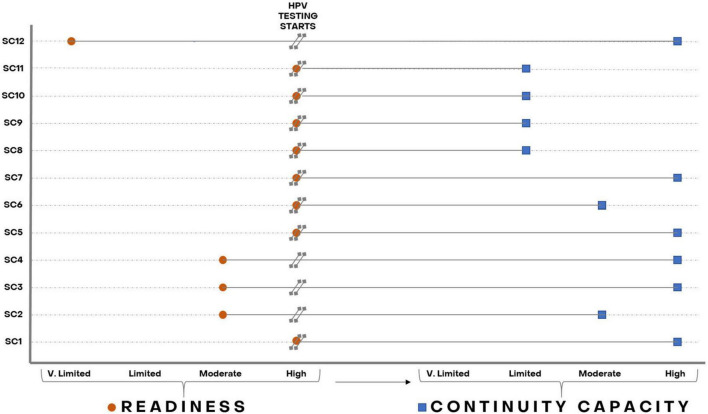
Laboratories’ readiness and continuity capacity to perform HPV testing throughout recruitment in the ESTAMPA study. Orange circles represent laboratories’ readiness and blue squares laboratories’ continuity capacity as assessed from the start of HPV testing up to December 2021.

Figure 3 further depicts the relation between readiness and continuity capacity for HPV testing. Two (SC1, SC5) of seven laboratories (SC1, SC5, SC6, SC8, SC9, SC10, and SC11) with *high readiness* also showed *high continuity capacity*, one (SC6) showed *moderate continuity capacity*, and the other four (SC8, SC9, SC10, and SC11) were not able to maintain good-quality HPV testing over time after an excellent start and showed *limited continuity capacity*. Among three laboratories with *moderate readiness*, one (SC2) kept *moderate continuity capacity* and two (SC3 and SC4) reached *high continuity capacity.* The two laboratories new to molecular testing (SC7, SC12) with subsequent *limited* and *very limited readiness*, respectively, achieved *high continuity capacity*.

### Recruitment targets and laboratories’ participation in national screening programs by level of continuity capacity

Table 3 details the recruitment targets and their attainment status at the end of the assessment period, as proxy of HPV testing volume over the study, and whether laboratories is currently part or is considered to become part of the national screening program by level of continuity capacity. Among six study centers whose HPV laboratories showed high continuity capacity, SC5 reached its recruitment target, SC12, that is still recruiting, has achieved 64% of the target, SC4 and SC7 did not reach their targets, and SC1 and SC3 exceeded them. SC4 stopped after recruiting 88% of its target because of difficulties with the uptake of screening for several months, while SC7 stopped at 67% mainly due to termination of the loan of the COBAS machine to the study team and emerging priorities of the clinical team. Both SC2 and SC6 with moderate continuity capacity achieved their targets. SC9 only targeted 500 women and stopped at 82% after a slow recruitment pace over time which worsened with COVID pandemic restrictions. The SC8 team experienced sudden change of political and organization authorities who caused important disruptions to screening activities with unexpected disassembling of the study clinic. The SC10 highly experienced research laboratory faced study leadership instability leading to poor coordination of screening and laboratory activities. In view of these political and leadership issues, recruitment stopped at 14 and 17% attainment for SC8 and SC10 respectively. The only laboratory with direct HPV testing problems was SC11, where inadequate maintenance of the HPV equipment led to 31% of screened women (*n* = 497) having invalid HPV results. Immediate correcting measures to guarantee the safety of participants (e.g., multiple attempts to repeat sampling, recalling women directly to colposcopy) were implemented in these three SCs.

**TABLE 3:** Recruitment targets per study center (as proxy of HPV testing volume) and inclusion of laboratories in national screening programs by level of continuity capacity.

Continuity capacity	SC/Laboratory	Recruitment up to December 2021		Laboratory in the national screening program?
		Target	Target attainment	
High	SC1	10,000	Target exceeded (110%)	Yes
	SC3	5,000	Target exceeded (127%)	No
	SC4	5,000	Stopped due to COVID. Target not attained (88%)	No
	SC5	2,000*	Target attained (> 99%)	No
	SC7	5,000	Stopped. Other clinical team priorities. Target not attained (67%)	Yes
	SC12	5,000*	Recruitment ongoing until December 2022, 64% of target attained.	No
Moderate	SC2	1,250*	Target attained	No
	SC6	10,000**	Target attained	Yes
Limited	SC8	5,000	Stopped. Multiple political issues affected screening and research activities including laboratory procedures. Target not attained (14%)	No
	SC9	500	Stopped due to COVID. Target not attained (82%)	Yes
	SC10	5,000	Stopped. Multiple leadership and coordination issues affected screening and research activities including laboratory procedures. Target not attained (17%)	Yes
	SC11	500	Recruited 497 women but 154 (31%) had invalid results. Target not attained (69%)	No

SC: study center. SC/Laboratory: laboratory performing HPV testing for each SC.

*Started with pilot targeting 500 women.

**Initial target of 5,000 increased to 10,000 due to strong local health authorities’ endorsement.

Five of the 12 laboratories are now or soon will be part of their national HPV-based screening program, including SC1 and SC7 with high continuity capacity, SC6 (moderate) and despite challenges during the study, SC9 and SC10 with limited continuity capacity based on their nature (national referral public health, highly experience research) and on the experience, increased installed capacity and lessons learnt throughout the study.

## Discussion

The WHO recommends screening women with a high-performance test, such as HPV DNA testing, twice by age 35 and 45, and encourages countries to move from cytology- or VIA- to HPV-based cervical screening programs. However, most cervical screening worldwide is done with cytology and transition to HPV-based cervical screening should be carefully planned. There are several important factors to consider, such as the size of target population, the screening approach, women’s acceptance and participation in HPV testing, local availability of HPV testing platforms (equipment, consumables), laboratory installed capacity, availability of trained personnel, quality assurance, and other operational aspects.

In our study, two main factors enabled successful performance of HPV laboratories as part of HPV introduction: the initial assessment of existing laboratory capacity and the regular support offered to participating laboratories throughout the study. The initial assessment identified limitations in the laboratory’s ability to perform HPV testing and helped laboratory teams to develop a road map (staff training, equipment, procurement plan) to address shortcomings before launching the study. In all laboratories, rapid training of new technicians, regardless of their role, was provided in order to minimize disruption of activities. The simple hands-on processing of both HPV tests used in the study (HC2 and COBAS) also helped train new personnel. The training was usually completed within a week, even where staff had no previous HPV testing experience.

The ESTAMPA study aims to evaluate triage techniques for HPV positive women and contribute to the implementation of HPV-based cervical cancer screening in Latin America. Two baseline cervical samples were collected using a cytobrush washed in vials containing PreservCyt medium (PC) to assess the performance of different molecular triage techniques. HC2 testing is incompatible with PC and uses its own collection medium (Digene sample collection medium, STM). This meant that study centers using HC2 had to prepare samples for testing using a conversion kit, adding on average 1.5 full working days per testing batch of 88 samples. Additionally, all laboratories (independently of the HPV test used) were requested to produce aliquots from both PC vials for future evaluation of molecular triage techniques. This process could take up to one full working day. Additionally, screened positives attended a colposcopy visit at which another sample for HPV testing was collected, and those not receiving treatment based on colposcopy and histology results were recalled at 18 months and had another HPV test, and aliquots were produced from samples once HPV tested. In real-life settings, laboratories may have more capacity for HPV testing as these extra research activities will not be required.

The average HPV positivity over time was 14.1%, with some fluctuations related to debut and end of recruitment and testing at each center along the study. We detected HPV positivity values below 10% or above 16% (the inspection threshold) in some centers over time. This triggered an immediate evaluation of the entire screening process by using the study centralized web-system, followed by a site visit to reinforce training and ensure safety of participants as needed. In most cases, low or high HPV positivity was usually associated with characteristics of the included study population. For instance, one center reported a consistently high positivity (>16%), most likely due to the recruitment of a referral population (women with previous abnormal smears attending colposcopy) (data not shown). Assessments suggested HPV testing problems only in one laboratory, where immediate evaluation of the HPV equipment and a plan to maintain good cleaning and calibration of laboratory equipment were requested. This laboratory could not comply with these measures and the study had to stop before reaching the recruitment target.

Readiness for HPV testing was measured ahead of starting recruitment, and study teams whose laboratories were not fully ready to start, took several months (up to 3 years) to deal with shortcomings and they only started recruitment and testing once they fulfilled *high readiness* requirements. Once testing started, laboratories faced challenges in achieving *high continuity capacity* for HPV testing. In some study centers, the recruitment rate was unexpectedly low at times, leading to several weeks of delay before enough samples for a whole testing batch were collected, sometimes leading to expiry of reagents before HPV testing and impacting the turnaround of HPV results. Corrective measures such as testing half batches were introduced; however, sometimes, even half-batch testing was not achievable, particularly during the COVID pandemic, and inevitably led to wastage of reagents. In addition, as HPV testing had not been rolled out in almost all participating countries during the study, collection medium and HPV testing reagents needed to be imported as local procurement was not possible. Local principal investigators had to quickly learn regulations to import unregistered products, logistics associated with customs clearance, and different aspects of the procurement process, including price negotiation and management of expiry and stock levels of consumables. Laboratories managers were encouraged to subscribe to international EQA schemes. Six laboratories had yearly subscription to EQA schemes before the start of the study as required by local policies. The study covered the first-year fees for other four laboratories, but only one of them managed to continue with subscriptions afterward. Fees for the remaining two laboratories were not provided by the study nor covered by the laboratory. The main reason for not ensuring funds for EQA, in addition to limited resources, was the lack of a “culture of quality”, as managers did not understand the importance of maintaining regular EQA not only for HPV testing but for other laboratory activities.

We did not observe any relationship between laboratory high continuity capacity and attainment and size of recruitment goals. Of six laboratories with high continuity capacity, two exceeded their original recruitment targets of 10,000 and 5,000 by 10% and 27%, one reached its target (2,000), one is still recruiting (already 64% attained) and two aiming to recruit 5,000 had to stop at 67% and 88% attainment. The two with moderate continuity capacity reached their targets (1,250 and 10,000); and none of the four showing limited continuity capacity completed recruitment, two originally targeted 500 and the other two 5,000 women. Of importance, independently of the level of continuity capacity, five laboratories will become or are already part of their national screening program. For instance, one laboratory with high continuity capacity that stopped recruitment because the HPV testing platform loan ended, and the equipment was retrieved by the manufacturer company is now part of the national program though using a different HPV testing platform but maintaining the laboratory staff trained on HPV testing for the study. The incorporation of these five laboratories into national programs demonstrates that all efforts in training, continued support, and engagement with and between local and regional stakeholders during the study, are certainly contributing to the implementation of HPV-based screening programs in Latin America.

Most difficulties faced by laboratories reflect the early days of the HPV testing market. Currently, more than 250 HPV tests are available on the market, although most of them are not yet adequately validated (14, 39). It is important that manufacturers invest in validating their tests in line with consensus requirements that ensure safe use in clinical settings (40, 41). Notably, several adequately validated tests can be run in small batches or even individual samples and include internal (per sample) controls that can facilitate monitoring testing accuracy (14). Such tests are ideal for scenarios where the target screening population is small, or reaching screening coverage will take a long time, or where access to care is difficult and screening uptake is limited.

The main strengths of our results are: (1) The diversity of laboratories where HPV testing was performed, contributing with valuable information on the multiple challenges that countries may face during transition to, implementation and scale-up of HPV testing in primary screening; (2) The use of regular feedback given by the central coordinating team and most importantly through exchange of experiences and lessons through the strong ESTAMPA study network, which facilitated installing a “culture of quality” in laboratories, and (3) The demonstration that readiness and continuity capacity are good indicators of how HPV implementation is ongoing in terms of laboratory management and quality of HPV testing, and can be assessed using simple checklists and questionnaires. On the other hand, two limitations of our study are related to these simple tools. First, information provided by laboratory researchers or managers may be prone to reporting bias, and second, continuity capacity was assessed based on responses to a single-time questionnaire. Nevertheless, in our study, these limitations were mitigated by completing and verifying information through monitoring visits to all teams and their participating laboratories over time. However, it should be noted that in the context of a screening program, regular monitoring visits may not be feasible and other strategies may be needed to complete implementation assessment.

The 12 laboratories participating in ESTAMPA were essential for study centers screening and overall study achievements, despite several of them being new to HPV testing or to its use in cervical screening and having faced challenges to adopt the technique into daily activities. The strong support provided by the study network facilitated overcoming most difficulties leading to 42,502 HPV screened women across 12 study centers. We have described here the process of introducing HPV testing in 12 laboratories across Latin America, we further plan to use data per HPV testing batch to evaluate and compare HPV performance across laboratories, over study visits (enrolment, colposcopy, 18-months follow-up) and over time.

## Conclusion

In summary, our assessments confirm that as *high readiness* is essential for successfully implementing HPV testing in laboratories; however, *high readiness* is not sufficient to guarantee HPV testing *high continuity capacity*. Several aspects to achieve this *high continuity capacity* should be considered:

(1) A “culture of quality” in laboratories and across the cervical cancer screening spectrum should be established, including regular training on SOPs, robust monitoring and quality assurance systems (with internal and external quality control measures) tailored to the installed capacity and local resources
(2) Using a friendly and tailored to local context database, if possible centralized and web-based, to allow regular monitoring of the overall screening process, including laboratory and clinical activities, and feedback for improvement
(3) Training on preparation of appropriate plans for HPV supplies procurement and stocking and on local regulations on products registration and importation of laboratory supplies should be provided to laboratory managers and leaders of cervical screening programs
(4) Strategies to guarantee reasonable turnaround of HPV results to women should be in place to ensure that any screened woman receives her HPV results and that screened positives receive adequate and timely treatment and follow-up
(5) Exchange of experiences and lessons learnt between multidisciplinary implementers from different settings and countries should be encouraged to apply most suitable implementation strategies according to context and define additional implementation research needs.

## Data availability statement

The original contributions presented in this study are included in the article/Supplementary material, further inquiries can be directed to the corresponding author.

## Ethics statement

The studies involving human participants were reviewed and approved by the Ethics Committee of the International Agency for Research on Cancer (IEC Project 12-27-A7), the Pan American Health Organization (PAHO) Ethics Committee and Ethical Committees in each of the study participating centers. The informed consent include details on the background, procedures of the study, risks and benefits, statement of confidentiality, specimen use and study staff to contact. The study is considered minimal risk as the procedures are standard practice in cervical cancer screening programs. The patients/participants provided their written informed consent to participate in this study.

## Authors contributions

MA and RH conceived the ESTAMPA study and are the principal investigators responsible for its overall conduction. MAP, AF, GS, LM, CT, LF, VV, GV, AC-V, GR, AC, and CW are the local principal investigators responsible for recruitment, clinical management, and data collection. MAP, AF, and GS conceptualized the laboratory aspects of the study and together with MLR, MH, LM, MRP, CT, PH-N, FD, JA-S, MZ, and VV coordinated all laboratory activities including training of new technicians as needed. JL, AP, MB, LM, PM, YC, RC, DC, LG, AR, MR, MLB, and LC performed HPV testing. JV and AB provided statistical support and together with MA, MH, MLR, EL, and AR centrally coordinated the study. MA, RH, NB, and SL contributed to the funding acquisition and local capacity building. MLR and MA wrote the manuscript. MH, AB, JV, SL, NB, and RH contributed to the finalization of the manuscript. All authors reviewed and approved the final version of the manuscript.
