# Supplementary material for: Implementing HPV testing in 9 Latin American countries: The laboratory perspective as observed in the ESTAMPA study
Source: Front Med (Lausanne). 2022 Nov 17;9:1006038. doi: 10.3389/fmed.2022.1006038 (PMC9714610; doi:10.3389/fmed.2022.1006038)
Supplement: Supplementary file 1 [file Data_Sheet_1.pdf]

LIST OF REQUIREMENTS TO IMPLEMENT HPV TESTING FOR ESTAMPA

1. LABORATORY FACILITY AND EQUIPMENT:

a) Basic settings
b) Equipment for HPV testing: COBAS/HC2
c) EQA for HPV testing
d) Refrigerator 4C to store HPV reagents
e) Freezer -20C to store aliquots
f) Freezer -70C to store the swabs

2. PERSONNEL TO:

a) Process samples for HPV testing,
b) Preparation of aliquots of cervical samples
c) Uploading results in the ESTAMPA database
d) Person in charge of monitoring and quality control of forms and database

3. PROCUREMENT

3.1 SAMPLES COLLECTION SUPPLIES:

a) PreservCyt:

In case IARC buys them:

Import permit of the PreservCyt vials if they are not registered in the country

It is necessary to contact a Transport Company that can deliver the HOLOGIC merchandise; it should be a company authorized to carry dangerous merchandise (The PreservCyt containing methanol) and has experience in the process of customs clearance

If the group buys locally HOLOGIC the reference needed are: PreservCyt (Ref: 70098-002) Cervex brush (Ref: 70101-001)

Recruitment Supplies needed: 2 Vials PC per woman

b) Cervex brushes: 2 per woman

3.2 HPV TESTING REAGENTS

a) Reagents for performing the HPV test
b) If CH2: Conversion kits (needed to process samples in PreservCyt) and capture kits (Qiagen).

3.3 CONSUMABLES FOR THE PRODUCTION OF ALIQUOTS:

a) 1.250 mL tips with filter (sterile) for producing aliquots, Dutsher Ref 134000
b) Cryovials, 2 mL Corning to aliquot cervical samples Ref Corning 430488 (Fisher 10418571)
c) 1 mL Nunc cryovials to aliquot serum, plasma and White cells, DutsherRef 55001
d) Cryoboxes cardboard storage + separator 100 positions, Dutsher Ref 39818+ Ref 39824
e) Sterile gloves

4. DATA ENTRY EQUIPMENT

a) Computer with internet Access for loading results to the online database ESTAMPA (https://screening.iarc.fr/scr/estampa/sec)

DOCUMENTATION:

a) Submission of protocol and Inform consent (IC) to a local scientific ethics committee
b) Approval of the protocol and the IC by the local scientific Ethics Committee
c) Research collaboration agreement signed with IARC
d) Establishment of a local samples transport system (if required)
