# Supplementary material for: Implementing HPV testing in 9 Latin American countries: The laboratory perspective as observed in the ESTAMPA study
Source: Front Med (Lausanne). 2022 Nov 17;9:1006038. doi: 10.3389/fmed.2022.1006038 (PMC9714610; doi:10.3389/fmed.2022.1006038)
Supplement: Supplementary file 2 [file Data_Sheet_2.pdf]

Questionnaire on laboratory operational aspects

1A About the test

**1A.**	¿Is HPV testing part of the health system?	YES/No
**1A.1**	Was the HPV test implemented for ESTAMPA?	YES/No
**1A.2**	When did the lab processing samples for screening started?	dd/mm/yyyy
**1A.3**	Does the lab run EQ controls?	YES/No
**1A.3.1**	What type of EQA is used?	1) CAP<br>2) Other?

1B About the laboratory's test processing capacity

	**Approximate number of samples in 1 month:**	**n**
**1B.1**	Received samples	
**1B.2**	Processed samples	
**1B.3**	Delivered results	
**1B.4**	Estimated time (days) for the availability of results	

1C About the Personnel who run the test

**1C.1**	Number of professionals, technicians and administrative staff assigned to the HPV Laboratory	

**Name and Surname**	**Position/Title**	**Dedication (full time or part-time)**	**Years of Experience in HPV lab.**	**Received training (Describe which one)**

**1C.2**	**Laboratory staff**	**YES/NO**

Questionnaire on laboratory operational aspects was based on the "Malbrán institute" questionary For HPV Testing Implementation

1C.3	Is the staff adequately trained?	
1C.4	Is the number of trained personnel adequate for the workload?	
1C.5	Are staff members familiar with protocols for sample collection and transportation?	
1C.6	Are staff members trained to advise other technicians and/or professionals?	

	**Personnel collecting the samples**	
1C.7	Who collects the HPV samples?	**a) Doctors (general practitioners, gynecologists)**<br>**(b) Nursing staff (technicians)**<br>**c) Bacteriologists, microbiologists**
1C.8	Did staff members receive training in cervical sampling?	**(a) Yes**<br>**(b) No**
1C.9	Did t staff members receive appropriate training on how to use the forms and fill in the information; emphasizing on the importance of the woman claiming the result and being followed-up?	**(a) Yes**<br>**(b) No**
1C.10	Are staff members taking the sample the same as the one who deliver the HPV result?	**(a) Yes**<br>**(b) No**<br>**If not, describe who delivers the result**
1C.11	Did the staff member who delivered the result receive appropriate training in communicating HPV results?	**(a) Yes**<br>**(b) No**

	About the sample collection	YES/NO
1C.12	When samples for HPV testing are taken, do you ask if antifungal creams, contraceptive gel, or vaginal hygiene products are present?	

1D. On the operating procedures and operation of the HPV laboratory

	Space	YES/NO
1D.1	Is the area used efficiently?	
1D.2	Is the configuration of the space adequate and follows good laboratory practices?	
1D.3	Does the laboratory have an air conditioning system?	
1D.4	Does the laboratory have a backup system in case of a power outage (UPS/generator set)?	
1D.5.	Are the enclosures adequately sealed to prevent dust particles from entering inside?	
1D.6.	Is the laboratory cleaned and well maintained?	

(1) if possible, provide photos of the laboratory, equipment, and reagents

(2) Consider equipment location

(3) Take into account lighting, countertop surface, accesses, etc.

	Laboratory supplies and reagents	YES/NO
1D.7	Do you have the basic supplies for daily work?	
	a. Disposable talc-free gloves	
	b. Absorbent paper	
	c. Self-adhering film	
	e. Pyrogenic water	
	f. chronometer	
	g. Material for the preparation of solutions	
	h. Disinfectant solution (isopropyl alcohol, ethanol)	
1D.8	Is the provision of basic supplies assured?	
1CD.9	Do you have the specific inputs and reagents for daily work?	

Questionnaire on laboratory operational aspects was based on the "Malbrán institute" questionary For HPV Testing Implementation

	a. Kits	
	b. Automatic pipettes	
	c. Tips with and without filter	
1D.10a	Are the provisions of specific reagents and inputs assured?	
1D.10b	Are the expiration dates of each of the reagents for HPV testing monitored?	

List the main reasons that hinder the supply of inputs (kits and consumables):

1)

2)

3)

	**Laboratory equipment**	**YES/NO**
1D.11	Is the number of instruments suitable for the workload?	
1D.12	Are the instruments in good condition?	
1D.13	Are there periodic checks on the operation of the instruments (Tº registration), calibration, etc.?	
1D.14	Do the instruments have a backup system in case of a power outage (UPS/generator set)?	

	**Storage equipment**	**YES/NO**
1D.15	Is the amount of equipment adequate for the workload?	
1D.16	to. Freezer -20°C	
1D.17	b. Freezer -70 °C	
1D.18	b. Refrigerators 4-8ºC	
1D.19	Is the equipment in good condition?	
1D.20	Are there periodic checks on the operation of the equipment (T° register)?	
1D.21	Does the equipment have a backup system in case of a power outage (generator set)?	

Questionnaire on laboratory operational aspects was based on the "Malbrán institute" questionary For HPV Testing Implementation

1D.22	Are the freezers (-20C and -70C) located inside the laboratory?	
1D.23	If the freezers are not in the laboratory, in which way and how often are the samples transported to the freezers?	
1D.24	Are there independent dedicated spaces for the storage of samples and reagents?	
1D.25	Were the samples stored at room temperature?	a) Yes<br>(b) No<br>If this is the case, mention for how long<br>_________
1D.26	Were the samples refrigerated between 2-8 °C?	a) Yes<br>(b) No<br>If this is the case, mention how much you time

1E. Management of results and electronic files

**1EA**	**About the staff entering the results**	**YES/NO**
1EA.1	Are staff members who enter the data into the database adequately trained?	
1EA.2	Is the number of staff trained adequate for the workload?	

1EA.3	**Equipment**	**YES/NO**
	Is the amount of equipment (computers, tablets, etc.) adequate for the workload?	
	Is the equipment in good condition?	
	Do you have an internet connection?	
	Does the equipment have a backup system in case of a power outage (UPS/generator set)?	

Questionnaire on laboratory operational aspects was based on the "Malbrán institute" questionary For HPV Testing Implementation

2. Integration of the HPV laboratory with the other laboratories of the service and transport of samples

**2A**	Is there a good communication between the screening laboratory and...?:	**YES/NO**
	a. Gynecology Service	
	b. Cytology Section	
	c. Pathological Anatomy Section	
	d. Hospital or recruitment center authorities	
	e. Authorities of the Program or Institution sponsoring the local project	
**2B**	Ensuring that the samples were transported from the recruitment center to the laboratory where the test is performed:	**YES/NO**
	a. Were they integrated into the health system and transported along with other clinical samples to be processed?	
	b. Was dedicated staff hired to transport the samples?	
	c. Was a member of the recruitment team in charge of transportation?	
**2C**	Was there an SOP to regulate the transport of the samples?	

3. Delivery of results to patients and follow-up

		**n**
**3A**	How long (days) on average the results were delivered to the patients after the collection of the samples	
3B.	Which clinician communicates the result to the patient:<br>1. Medical<br>2. Nurse<br>3. Other (specify):	

Questionnaire on laboratory operational aspects was based on the "Malbrán institute" questionary For HPV Testing Implementation

3C. In case of positive result:	**YES/NO**
a. Does the patient receive recommendations on how to continue his/her treatment?<br>1. Yes<br>2. No	
b. Does the patient receive psychological support?<br>1. Yes<br>2. No	
c. Is it established to which health service or clinical institution HPV+ women are referred for a colposcopy?	
d. Which referral mechanism is used?	(a) Letter<br>b) Phone call<br>(c) Another, which one?
e. Is there a form enabling communication between the HPV testing service and the colposcopy referral center?	
f. Are woman's contact records kept to help reducing missed spells during follow-up?	
g. Is there a registration system that clearly identifies HPV-positive women and allows for prioritization for follow-up?	a) Yes<br>(b) No<br>If yes, describe which one and how it works
h. Is there a mechanism to contact and follow-up with women who missed a scheduled appointment?	a) Phone call<br>b) Letter of invitation<br>(c) Personal visits<br>(d) Other, describe which

1. Implementation of HPV testing at a general level

Questionnaire on laboratory operational aspects was based on the "Malbrán institute" questionary For HPV Testing Implementation

		YES/NO
4A	Are there plans to implement HPV testing in the country?	
4B.	Is there a local health system that can provide adequate clinical management of positive patients?	
4C.	What would be the estimated cost of the test?	

Questionnaire on laboratory operational aspects was based on the "Malbrán institute" questionary For HPV Testing Implementation
