# Supplementary figures and images for: Implementing HPV testing in 9 Latin American countries: The laboratory perspective as observed in the ESTAMPA study
Source: Front Med (Lausanne). 2022 Nov 17;9:1006038. doi: 10.3389/fmed.2022.1006038 (PMC9714610; doi:10.3389/fmed.2022.1006038)

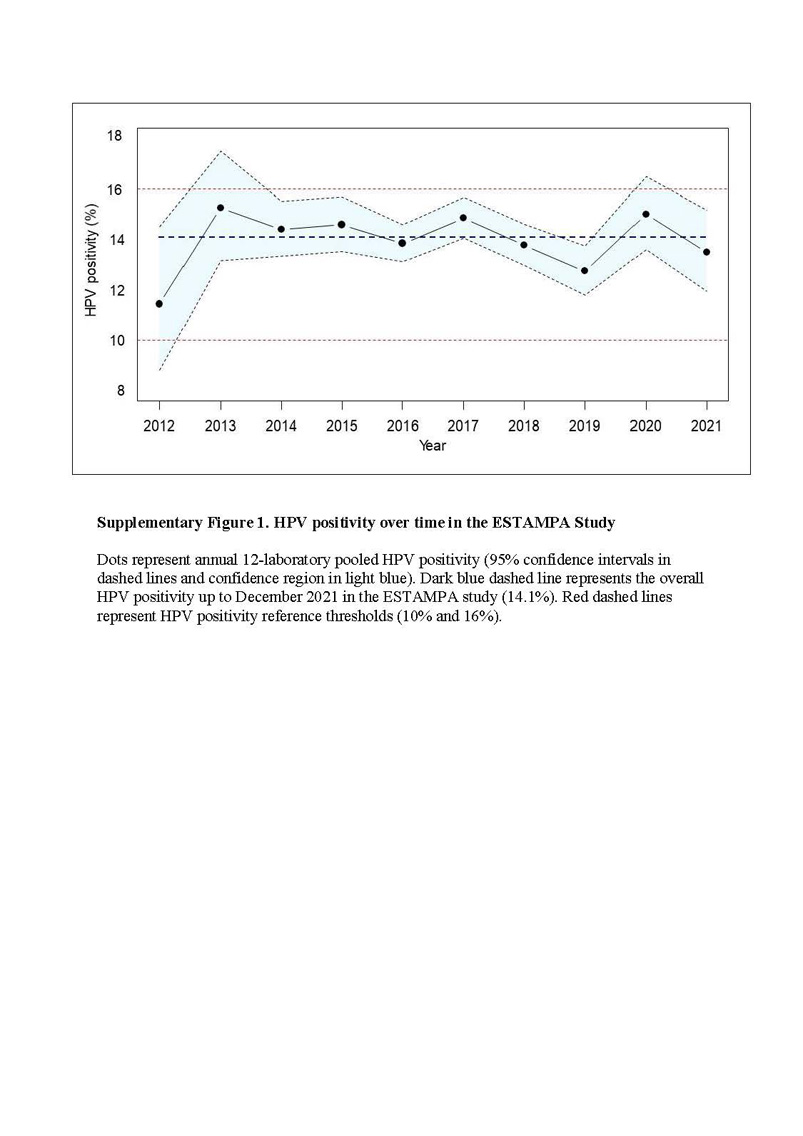

Supplement: Supplementary file 3 [file Image_1.jpg]
